# Radiotherapy waiting time in Northern Nigeria: experience from a resource-limited setting

**DOI:** 10.3332/ecancer.2020.1097

**Published:** 2020-09-04

**Authors:** Nuhu Tumba, Sunday Adeyemi Adewuyi, Kelechi Eguzo, Adeniyi Adenipekun, Rasaaq Oyesegun

**Affiliations:** 1 Department of Radiology, Division of Radiation/Clinical Oncology Bingham University Teaching Hospital, Jos 930214, Nigeria; 2 Department of Radiotherapy & Oncology Ahmadu Bello University Teaching Hospital, Zaria 810105, Nigeria; 3 Department of Obstetrics & Gynaecology, University of Saskatchewan, S4N 2B7, Canada; 4 Department of Radiation Oncology, University College Hospital, Ibadan 200284, Nigeria; 5 Department of Radiotherapy & Oncology, National Hospital Abuja 900211, Nigeria

**Keywords:** waiting time, radiotherapy, resource-limited-setting

## Abstract

**Background:**

Access and availability of radiotherapy treatment is limited in most low- and middle-income countries, which leads to long waiting times and poor clinical outcomes. The aim of our study is to determine the magnitude of waiting times for radiotherapy in a resource-limited setting.

**Methods:**

This is a retrospective cohort study of patients with the five most commonly treated cancers managed with radiotherapy between 2010 and 2014. Data includes diagnosis, patients’ demographics and treatment provided. The waiting time was categorised into intervals (1) between diagnosis and first radiation consultation (2) First consultation to radiotherapy treatment (3) Decision-to-treat to treatment and (4) Diagnosis to treatment.

**Results:**

A total of 258 cases were involved, including cervical (50%; 129/258), breast (27.5%; 71/258), nasopharynx (12.8%; 33/258), colorectal (5%; 13/258) and prostate cancers (4.7%; 12/258). Mean age was 48 (±12.9) years. Treatment with radical intent comprised 67% (178/258) of cases, while 33% (80/258) had palliative treatment. The median time from diagnosis to first radiation consultation was 40 (IQR 17–157.75) days for all the patients, with prostate cancer having the longest time – 305 days (IQR 41–393.8). The median time between the first radiation oncology consultations and first radiotherapy treatment was 130.5 (IQR 14–211.5) days; cervical cancer patients waited a median of 139 (IQR 13–195.5) days. The median time between diagnosis and first radiotherapy for breast cancer patients was 329 (IQR 207–464) days, compared to 213 (IQR 101.5–353.5) days for all the patients.

**Conclusion:**

The study shows that waiting time for radiotherapy in Nigeria was generally longer than what is recommended internationally. This reflects the need to improve access to radiotherapy in order to improve cancer treatment outcomes in resource-limited settings.

## Background

Radiotherapy is an important component of modern cancer treatment, with an estimated 50%–60% of all cancer cases receiving it during the course of their treatment. Yet access to this vital modality of cancer treatment is limited or even absent in most low- and middle-income countries (LMICs), especially in sub-Saharan Africa. It is estimated that 80% of the world’s cancer burden is in LMICs, but only 5% of the world’s radiotherapy resources are in LMICs. Therefore, about 90% cancer patients who need radiotherapy have no access to it and those who do have access have to wait a long time to receive treatment [1–6].

The fundamental reason for long waiting times for radiotherapy is the increasing demand for radiotherapy due to raising cancer incidence in LMICs as shown by epidemiological studies and the recent GLOBACAN 2018 reports published by the International Agency for Research on Cancer. This rising incidence has not been matched with a proportional expansion in radiotherapy infrastructure and the current capacity does not match the existing needs and unless this is addressed urgently, the situation will even be more critical in the next decade. Other factors include insufficient radiotherapy professionals (radiation oncologist, medical physics, therapy radiographers and mould room technicians), increasing complexity in the treatment process, poor maintenance which increases machine downtime and frequent strike action by health care professionals to demand for better working conditions and remuneration [7, 8].

Several clinical studies have shown the adverse effects of delay in initiation of radiotherapy treatment. Long waiting time or delayed radiotherapy have been shown to increase the risk of local recurrence and decrease in overall survival in certain clinical settings [9–14]. Jansen *et al* [15] have estimated a decreased local control rate of 8% per month of delay in a study of head and neck cancers. Apart from these obvious poor clinical outcomes, delayed radiotherapy exacerbates the anxiety that arises from a cancer diagnosis in patients and their caregivers. Losses of productivity and income as well as increased medical expenditure are often overlooked consequences of long waiting times [16, 17].

Many studies have examined the problems of waiting, especially in high-income countries, but few studies have examined radiotherapy waiting time in resource-limited settings.

The aim of our study is to determine the magnitude and the variation of waiting time across different categories of cancer patients.

## Materials and methods

### Study setting

The Ahmadu Bello University Teaching Hospital is situated in Zaria, Kaduna state with an estimated population of 8,252,366. However, we receive patients from all over Nigeria, usually when there is breakdown of machines in the other centres and from the neighbouring countries of Chad, Cameroun and Niger.

The Radiotherapy & Oncology Department, Ahmadu Bello University Teaching Hospital is the first radiotherapy facility in northern Nigeria. It was established in 1995 and has a low dose rate brachytherapy machine which mainly treats cervical cancers. The cobalt-60 tele therapy machine was commissioned in April 2000, and it is currently one of two megavoltage machines in the northwestern region (the other centre being at the Usman Dan Fodio University Teaching Hospital, in Sokoto State) comprising seven states with an estimated combined population as of 2016 of 48,942,347 [18, 19].

The department is also one of the four institutions for residency training programme in radiation and clinical oncology in the country. The others are Lagos University Teaching Hospital, University College Hospital Ibadan and National Hospital, Abuja.

Typically, new and stable patients present through the new patient clinics on Mondays or Thursdays during which they are seen and given laboratory investigations and imaging studies to do. They are subsequently reviewed by the consultant-in-charge who takes the decision for treatment. Emergencies are seen every day either from the wards or through the accident and emergency unit of the hospital.

Radiotherapy treatments are done every weekday, Monday to Friday between 8:00 am and 4:00 pm local time. No treatments are carried out in out-of-work-hours, weekends and public holidays. This is essentially due to a lack of adequate staff. Table 1 shows the current staff distribution [20, 21].

### Study population

A total of 2.902 patients were referred to the department during the period under review (January 2010–December 2015) with an annual average of 580 patients. We included patients with histologically confirmed cancer, complete medical and treatment records treated with at least one fraction of radiotherapy between 2010 and 2014.

A total of 342 met our inclusion criteria; however, we included only tumour sites with frequency of more than ten (which represent the commonly treated tumour sites in the department) for analysis leading to a total of 258 cases. This is to enable reasonable comparison between tumour sites.

This study did not include patients treated with brachytherapy because it is only offered to patients with cervical cancer.

Emergency treatment is given to any patient presenting with an oncological emergency, defined as any acute, potentially life-threatening event, either directly or indirectly related to a patient’s cancer or its treatment. Examples includes tumour haemorrhage, severe tumour pain and bone metastasis with imminent cord compression.

### Design and data collection

This is a retrospective cross-sectional study design. Data on sociodemographic variables and waiting time were collected using a pre-tested structured data collection form. We defined four dates as follows:

1. Date of diagnosis defined as date the pathology report was signed by the pathologist
2. Date of first consultation defined as the date patient was first seen at the radiation oncology clinic. This is when patients are seen and given imaging and laboratory test to do.
3. Date of review/decision-to-treat. This is when patients are reviewed with imaging and laboratory reports and the decision to treat is made during this visit
4. Date of first radiotherapy treatment. This is the date of first treatment.

**Figure 1.**
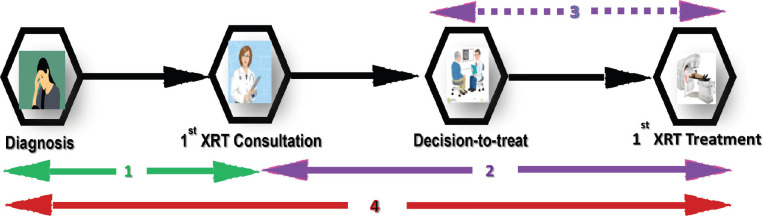
Patient pathway from diagnosis to treatment in the radiotherapy waiting time study 2010–2014.

Four time intervals were considered as shown in Figure 1 (Patients Care Pathway). The time was measured in weeks.

1. Interval between date of diagnosis to 1st consultation (WT1)
2. Interval between 1st consultation and 1st radiotherapy treatment (WT2)
3. Internal from decision-to-treat to 1st radiotherapy treatment (WT3)
4. Interval from diagnosis to radiotherapy treatment (WT4)

(XRT = Radiotherapy)

### Statistical analysis

Data were analysed using SPSS version 25. Descriptive statistics was computed for all the variables. Waiting time was measured as median, interquartile range and the 90th percentile. Because it is a continuous but skewed distribution, we used the Kruskal–Wallis test to compare median waiting time between groups. Alpha value of *p* ≤ 0.05 is taken as significant.

### Ethical consideration

The study was approved by the Ahmadu Bello University Teaching Hospital ethical review committee.

## Results

A total of 258 cases were reviewed, mean age was 48 (±12.9) years, males account for 37 (14.3%) while females account for 221 (85.7%). Cervical cancer accounts for 50% of cases followed by breast cancer (27.5%). Other patients’ characteristics are shown in Table 2.

Tables 3 and 4 show a summary of the waiting time measures, including the median, interquartile range based on cancer type, treatment intent, waiting list and stage of disease.

The median time from diagnosis to radiation oncology consultation (WT1) is 6 (IQR 2–30] weeks with 90% of cases being seen within 43 weeks (≈10 months) after diagnosis. There is significant variation in the median time to radiation oncology consultation across cancer sites (*p* value = 0.000) with cervical cancer being delayed the least with a median time of 4 (IQR 2–8) weeks while prostate cancer was delayed for 44 (IQR 6–56) weeks.

The median time from radiation oncology consultation to radiotherapy (WT2) is 19 (IQR 2–22) weeks which varies significantly across the cancer sites/diagnosis (*p* value = 0.000). prostate being the least with a median of 1 (IQR 0-8] weeks while nasopharynx was 28 (IQR 15–37) weeks. 90% of cases waited for 91weeks.

The decision-to-treat to 1st radiotherapy (WT3) is the interval from when patients imaging and laboratory reports are reviewed by the physician-in-charge and the decision for treatment is taken. The median time for all cases is 16 weeks (IQR 1–28). The longest duration is seen in nasopharyngeal carcinomas 25 weeks (IQR 14–33), while prostate cancer has a median time of 1week (IQR 0–7). The variation across the cancer site is statistically significant (p value = 0.000).

The overall time from diagnosis to 1st radiotherapy treatment shows the total time spent waiting for radiotherapy treatment. Median time is 30 weeks (IQR 15–51) – those with breast cancer waited the longest with a median time of 47 weeks (IQR 30–66) and colorectal cancer patients waited the least with a median time of 9 weeks (IQR 5–26).

Table 5 shows the proportions of cases meeting the best-practice-target as recommended in the literature for radical and palliative treatments.

Table 6 shows proportions of delay beyond 3, 6 and 12 months, respectively. Only 27.5% and 43% of cases were treated within the recommended 30 days and 10 days for radical and palliative radiotherapy, respectively.

Table 7 shows a comparison of the various waiting time intervals between the different categories of patients. There is no significant difference between males and females across all the different time intervals. However, there are some variations by diagnosis/site, stage, treatment intent and waiting list status

## Discussion

The timeliness of cancer treatment is of essence and affects its outcomes as shown by several clinical and experimental studies. This is particularly important in LMICs where access and availability are limited, with 70%–80% of patients presenting with advanced disease and up to 70% require radiotherapy [1, 6, 22, 23].

Our study describes cardinal points of interest in the radiotherapy treatment process; diagnosis, consultation and decision-to-treat and treatment. It also represents different sets of patients, provider and systemic factors that influence delay at each point

In our study, the median interval between diagnosis to first radiation oncology consultation is about 6 weeks. This represents the delay in referral by the clinician who made the diagnosis and is influenced by his/her experience, knowledge of the benefits, indications and sequence of radiotherapy relative to other treatment modalities. The delay in presentation to the radiotherapy by the patient even after diagnosis could be influenced by the factors, such as ignorance, lack of finance and use of complementary and alternative medicines. Furthermore, systemic factors such as lack of clearly defined referral protocols, clinical pathways and non adherence to clinical guidelines which leads to poor care coordination and unnecessary delays. Our findings are similar to a study by Lohlun *et al* [9] in South African patients with cervical cancer where patients delayed for about 5 weeks from referral to radiation oncology consultation. But it is more than The European Board and College of Obstetrics and Gynaecology recommendation of 2 weeks from referral of suspected or proven gynaecological cancer to consultation [24].

This component of delay has been significantly reduced in other health systems by setting benchmarks and a target time within which referral to a specialist is done. Accelerated and dedicated pathways for referrals are also established to reduce unnecessary delays [25–28].

Although this component of the delay is beyond the control of the radiotherapy facility or the radiation oncologist, it contributes to the overall waiting time and adds to the cumulative negative effect on treatment outcome.

There is also some difference between the different tumour sites with prostate cancer delaying more than cervical cancer. These variations may be due to biological and clinical features of these tumours and the referring clinician’s disposition to it. Cervical cancer causes bleeding with associated foul vaginal discharge. The fear and anxiety associated with blood and the shame and embarrassment of an offensive vaginal discharge not only puts pressure on the clinician to refer quickly, it is also a concern for the patient to present early. Prostate cancer is generally insidious in the onset of symptoms and patients usually commence their treatment with the urologist. They are referred only when there are indications for radiotherapy and this is when disease is castrate-resistant and or metastatic.

The time from first radiation oncology consultation to first radiotherapy treatment (WT2) includes the time spent undergoing these laboratory investigations after initial consultation and decision-to-treat and treatment interval (WT3) which is a subset of the interval between consultation and treatment (WT2) as shown in Figure 1. The interval shows the real waiting time for radiotherapy because it starts when the patients have been fully evaluated and the decision to treat is made and excludes the time spent during evaluation. Although it can be argued that the primary standard of care for cancers like cervix and nasopharyngeal is radiotherapy, especially when diagnosed at an advanced stage and therefore waiting time starts at diagnosis. Both intervals reflect availability of diagnostic and radiotherapy treatment infrastructure and manpower. The current status of radiotherapy facilities in Nigeria is largely due to lack of planning and steady investment, and partly due to huge initial capital investment required for equipment, infrastructure and training. Although Nigeria is the most populous country in Africa, it has only 0.05 megavoltage machines per million people as opposed to 4 per million people as recommended by the IAEA. This is far below Egypt and South Africa where 60% of Africa’s radiotherapy resources are [3, 5, 6, 20, 22, 29–32].

The IAEA recommends 1 radiation oncologist per 250–300 patients. Nigeria has currently less than 100 radiation oncologists and an estimated 100,000 new cases of cancer every year and still has a great deficit of megavoltage machines and radiation oncologists. This will require sustained and incremental investment over the next decade [3, 5, 21, 30–36].

The total median waiting time from diagnosis to treatment (WT4) is significantly long (about 8 months). This period is long enough to cause tumour stage migration (i.e. progression from a lower stage to more advanced stage) which may change the treatment intent from curative to palliation and the waiting list status from routine/elective to emergency. This explains why treatment outcomes in LMICs are poor and made even worse with more than 70% of patients already presenting with locally advanced or metastatic disease.

Our result is lower than 12.2 months reported from University College Hospital in Ibadan Nigeria. This may be due to the small sample size of the study. It is unacceptably long when compared with similar studies in High Income Countries (UK and Nova Scotia in Canada)[25, 30].

Such prolonged waiting times will also cause significant anxiety for both patients and their care givers, loss of productivity and additional health expenses.

Taking a cut-off point of ≥ 3 months as delay from diagnosis to treatment (WT4), our study shows that 77% (Table 6) of our patients experienced delays in receiving radiotherapy and more than half (59%) were delayed for more than 6 months. This is more than 50.4% of patients reported from Botswana with a median time from diagnosis to treatment of approximately 3 months 91 (63–238) days. However, this study included all treatment modalities and the Botswana government covers all or subsidizes some of the costs of cancer treatment which may be responsible for the shorter delay. Delay may be pronounced in countries like Malawi where radiotherapy facilities are not available and patients have to travel abroad for treatment [37–39].

There is no significant difference between males and females across all the different time intervals., (WT1, WT2, WT3 and WT4). However, the interval WT2 and WT3 vary significantly across all cancer sites by stage, treatment intent or by the waiting list status. Advanced and metastatic diseases are less likely to delay because they are likely to be treated with palliative intent or as emergencies. These factors reflect the clinical decision-making process in the choice of treatment in oncology in general but particularly in radiotherapy. Palliative and emergency treatment planning are easier, faster, and given with hypo fractionation while curative/radical treatments require meticulous planning and takes time. This is illustrated by the fact that only about a quarter of radical treatments were done within 30 days and about half of palliative treatments were done within the 10 days as recommended in the literature. The proportion of palliative cases treated is more than radical treatments.

Our study has established that although patients may delay presenting for radiotherapy for reasons such as fear, travel distance and socio-economic factors, greater delay is experienced by those who made it to the radiotherapy facility due to inadequacy of equipment and manpower. It is, therefore, safe to propose that the triad of late stage-at-diagnosis, delay in seeing a specialist after a cancer diagnosis and the delay in starting treatment are the main reasons why the outcomes such as survival are abysmally low in most resource limited settings.

The results of our study have several implications for current and future planning, expansion and investment in radiotherapy services at local and national levels in Nigeria and other LMICs in Africa and beyond.

First: at the local level, delay in receiving radiotherapy is unlikely to improve without expansion and upgrade of the current equipment and increase in number and training of current radiotherapy professionals. This is necessary given the fact that the current equipment is about 20 years since its commissioning in April 2000 and it is time to change it. The staff presently cannot run more than a shift due to inadequacy especially the therapy radiographers and medical physics. Of the five radiation oncologists, three are in the professorial cadre with additional academic and administrative responsibilities. There is a need to increase this number for adequate service delivery. At the national level, there is a need for long-term planning and investment, improvement in procurement processes to include training and maintenance contracts and equitable geographical spread across the country. There is also a need for improved working conditions, remuneration and other incentives for healthcare workers particularly radiotherapy professionals to avoid the brain-drain phenomenon that is rampant across the country and the continent at large.

Secondly: systemic factors such as poor or absence of radiotherapy referral protocols, appropriate use of clinical guidelines and pathways should be integrated into the national cancer control policy to reduce delay in referrals among cancer care service providers. The introduction of functional multi-disciplinary teams into our practice will reduce poor coordination and reduce delays significantly, this has been shown to reduce delay by up to 50% in Botswana [3, 6, 8, 20, 40].

Lastly: radiotherapy is the most cost effective and needed modality of treatment in our current setting where an estimated 70% of all cancer patients will require it as part of their treatment. However, it is expensive for individual patients and their families because of out-of-pocket payments. At present, the national health insurance scheme coverage is less than 5% of the population and does not cover radiotherapy treatments. Including it will reduce financial burden and delays due to lack of finance for those covered by the scheme. For the majority not covered by the scheme, a kind of government subsidy or government bearing the cost of radiotherapy as is practiced in Botswana may be an appropriate strategy [30, 37, 39].

### Limitations

This is a retrospective audit of patients records and the quality of data in such a study design is associated with missing and incomplete information. However, we deliberately chose dates and intervals that are routinely documented.

## Conclusion

This study shows that radiotherapy waiting time in LMICs like Nigeria is causing a lot of delay in receiving radiotherapy and is longer than international best-practice targets. Urgent steps need to be taken to address the problems of infrastructure, insufficient radiotherapy professionals, poor care coordination and out-of-pocket payments to minimise the delays so that they are as short as reasonably achievable.

## Conflicts of interest

All authors declared that they have no conflicts of interest.

## Funding

There was no funding support for this study.

## Figures and Tables

**Table 1.** Current staff strength and distribution in the Radiotherapy & Oncology Department, Ahmadu Bello University Teaching Hospital, Zaria.

Professional	Number
Radiation & Clinical Oncologist	5
Medical Physicist	2
Therapy Radiographer	2
Oncology Nurse	4
Mould Room Technician	2
**Total**	**15**

**Table 2.** Characteristics of cases in the radiotherapy waiting time study, Zaria 2010–2014.

Characteristics		Frequency	Percentage
Sex	Male	37	14.3
	Female	221	85.7
Stage	Early	68	26.4
	Locally advanced	171	66.3
	Metastatic	19	7.3
Treatment intent	Radical	178	67.0
	Palliative	80	33.0
Waiting list	Routine/Elective	200	77.5
	Emergency	58	22.5
Diagnosis/site	Cervix	129	50.0
	Breast	71	27.5
	Nasopharynx	33	12.8
	Colorectal	13	5.0
	Prostate	12	4.7

**Table 3.** Median and interquartile range in weeks in the radiotherapy waiting time study, Zaria.

Waiting time (Weeks)	Cancer type, median and interquartile range					Total
	Cervix	Breast	Nasopharynx	Colorectal	Prostate	
WT1	4 (2–8)	24 (9–42)	5 (3–14)	9 (3–14)	44 (6–54)	6 (2–30)
WT2	20 (2–28)	18 (3–33)	28 (15–37)	2 (0–9)	1 (0–8)	19 (2–22)
WT3	17 (1–26)	13 (1–30)	25 (14–33)	1 (0–8)	1 (0–8)	16 (1–28)
WT4	25 (8–38)	47 (30–66)	34 (22–69)	9 (5–26)	47 (6–109)	30 (15–51)

**Table 4.** The median and interquartile range in weeks of radiotherapy waiting time across treatment intent, waiting list and stage, Zaria 2010–2014.

Wait time (weeks)	Treatment intent		Waiting list		Stage		
	Radical	Palliative	Routine	Emergency	Early	Advanced	Metastatic
WT1	6 (3–21)	6 (2–33)	6 (2–21)	6 (2–31)	5 (2–13)	6 (2–21)	24 (4–101)
WT2	21 (4–31)	2 (0–25)	22 (3–32)	2 (0–21)	23 (6–34)	18 (2–29)	1 (0–2)
WT3	20 (2–29)	1 (0–20)	20 (2–30)	1 (0–17)	22 (5–33)	13 (1–26)	0 (0–1)
WT4	32 (20–48)	24 (6–55)	32 (19–51)	22 (6–48)	35 (23–49)	29 (13–48)	41 (10–111)

**Table 5.** Proportion of cases meeting best-practice-target in the radiotherapy waiting time study, Zaria 2010–2014.

Best-practice-target	Cervix	Breast	Nasopharynx	Colorectal	Prostate
Proportion of patients treated within 30 days from decision-to-treat to first radical radiotherapy (*n* = 178)	26**%**	31%	11%	57%	100%
Proportion of patients treated within 10 days from decision-to-treat to first palliative radiotherapy (*n* = 80)	51%	63%	17%	67%	67%

**Table 6.** Delay in days and proportion (%) of patients seen within 3 months and delay of >3, 6 and 12 months for all cancer diagnoses/site in the radiotherapy waiting time study, Zaria 2010–2014.

Intervals	Diagnosis-to consultation (WT1)	Consultation-to Radiotherapy (WT2)	Decision-to-Treat to Radiotherapy (WT3)	Diagnosis-to Radiotherapy (WT4)
Median (IQR) days	40 (17–155)	131 (14–212)	131 (14–212)	213 (102–354)
% of patients seen within 3 months	65%	46%	48%	23%
% of patients with >3 months delay	12%	23%	24%	18%
% of patients with >6 months delay	13%	24%	23%	35%
% of patients with >1 year delay	10%	7%	7%	24%

**Table 7.** Comparisons across the different categories of cases in the radiotherapy waiting time study, Zaria 2010–2014.

Categories	p values across intervals			
	WT^1^	WT^2^	WT^3^	WT^4^
Sex	0.176	0.912	0.705	0.370
Stage	0.017	0.000	0.000	0.127
Treatment intent	0.841	0.000	0.000	0.073
Waiting list status	0.576	0.000	0.000	0.011
Diagnosis	0-000	0-000	0-000	0-000
